# A Lightweight Protocol for Secure Video Streaming

**DOI:** 10.3390/s18051554

**Published:** 2018-05-14

**Authors:** Algimantas Venčkauskas, Nerijus Morkevicius, Kazimieras Bagdonas, Robertas Damaševičius, Rytis Maskeliūnas

**Affiliations:** 1 Department of Computers Science, Kaunas University of Technology, Studentu 50-212, LT-51368 Kaunas, Lithuania; nerijus.morkevicius@ktu.lt (N.M.); kazimieras.bagdonas@ktu.lt (K.B.); 2 Department of Software Engineering, Kaunas University of Technology, Studentu 50-212, LT-51368 Kaunas, Lithuania; robertas.damasevicius@ktu.lt; 3 Department of Multimedia Engineering, Kaunas University of Technology, Studentu 50-212, LT-51368 Kaunas, Lithuania; rytis.maskeliunas@ktu.lt

**Keywords:** information security, computer networks, steganography, the Internet of Things, fog computing, cryptography

## Abstract

The Internet of Things (IoT) introduces many new challenges which cannot be solved using traditional cloud and host computing models. A new architecture known as fog computing is emerging to address these technological and security gaps. Traditional security paradigms focused on providing perimeter-based protections and client/server point to point protocols (e.g., Transport Layer Security (TLS)) are no longer the best choices for addressing new security challenges in fog computing end devices, where energy and computational resources are limited. In this paper, we present a lightweight secure streaming protocol for the fog computing “Fog Node-End Device” layer. This protocol is lightweight, connectionless, supports broadcast and multicast operations, and is able to provide data source authentication, data integrity, and confidentiality. The protocol is based on simple and energy efficient cryptographic methods, such as Hash Message Authentication Codes (HMAC) and symmetrical ciphers, and uses modified User Datagram Protocol (UDP) packets to embed authentication data into streaming data. Data redundancy could be added to improve reliability in lossy networks. The experimental results summarized in this paper confirm that the proposed method efficiently uses energy and computational resources and at the same time provides security properties on par with the Datagram TLS (DTLS) standard.

## 1. Introduction

**Figure 1:**
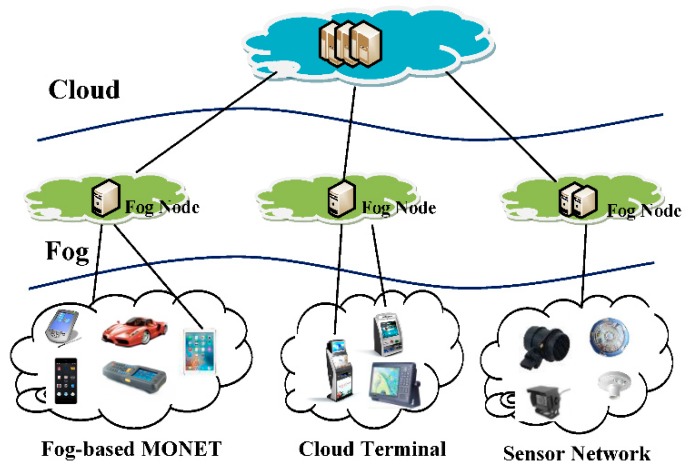
Three-layer fog computing architecture [5].

The emerging Internet of Things (IoT) introduces many new challenges that cannot be adequately addressed by today’s cloud and host computing models alone [1,2]. The most important challenges include (i) stringent latency requirements, (ii) network bandwidth constraints, (iii) device resource constraints, (iv) uninterrupted services with intermittent connectivity to the cloud, and (v) new security challenges. A study proposed by HP Fortify claims that 70% of the most commonly used IoT devices contain security vulnerabilities [3]. On the way to addressing these technological and security gaps, the IoT will require a new architecture known as fog computing [4], that distributes computing, control, storage, and networking functions closer to end user devices. Fog computing can be presented as a three-layer hierarchical architecture: Cloud-Fog-End Devices (Figure 1).

Fields of application for the IoT, as well as for fog computing applications, can be divided into the categories of environmental monitoring, smart cities, smart business/inventory and product management, smart homes/smart building management, health-care and security, and surveillance [6]. The fog computing security challenges and the possible solutions to these were thoroughly researched in articles by Stojmenovic, Roman, Tran et al. [6,7,8]. Some of the security challenges in the ”Fog-End Device” layer are authentication, rogue fog nodes, network security, secure data storage and secure and private data computation in the fog node, privacy, access control, and intrusion detection [6,9].

Existing cyber security solutions for today’s internet, designed primarily for protecting enterprise networks, data centers, and consumer electronics, have focused on providing perimeter-based protections. This existing security paradigm will no longer be adequate for addressing many of the new security challenges in fog computing. In examining the suitability of existing security solutions for fog computing, we see that these solutions are mainly intended for the “Fog-Cloud” layer. It is difficult to adapt them for the “End Device-Fog Node” layer, because the end devices have limited computation and energy resources, the environment is heterogeneous and distributed, and wireless communications have limited bandwidth. The most common security issues for the “End Device-Fog Node” layer are privacy concerns, insufficient authorization, lack of communications encryption, and inadequate software protection.

Many applications of the IoT, as well as of fog computing, will require the streaming of data from end devices to fog nodes and to the cloud. Data streams are mainly generated by end devices, including sensors, video cameras, smart phones, vehicles, portable devices, controls, and so forth [10]. Secure data streaming requires the fulfilment of three key security goals: confidentiality, integrity, and authentication [11]. Implementation of these goals in fog computing is the issue, especially in the “End Device-Fog Node” layer, because the end devices have limited computation and energy resources and network bandwidth constraints.

Wireless real-time video sensing applications make up a large part of IoT applications, and have become a relevant research topic in the last few years [12,13]. Video capable devices and applications include surveillance security cameras, smart traffic cameras, transit vehicles, house monitors, and more. In general, IoT security threats are inherent in video data streaming applications. However, video data streaming security needs are very difficult to fulfill because it is necessary to evaluate the specific requirements which include: real-time content requires enhancement of the delivery latency; the possibility of content broadcasting; confidentiality; nonrepudiation (including timestamping) and continuous authentication (CA) of the data stream source and content with limited computation; energy resources of the end devices; limited network bandwidth; and the need to resume secure data streaming after transmission interruptions [1,13,14].

This paper proposes a lightweight approach for continuous authentication of video data sources and streams with end devices with limited resources in the layer “End Device-Fog Node”.

The rest of the paper is organized as follows: related work is discussed in Section 2. The proposed covert channel-inspired secure streaming protocol is described in Section 3. In Section 4, the evaluation, experimental set-up, and experiments are presented. Finally, the paper is concluded in Section 5.

## 2. Related Work

There are several methods to provide data streams with security, of which cryptography, steganography, covert channels, and digital watermarking are the most commonly used methods. These methods can be implemented in different layers of the TCP/IP protocol stack.

Data streaming protocols are implemented using various transport protocols. Fairhurst et al. [15] described protocols which are the background for determining a common set of transport services—the Transmission Control Protocol (TCP), User Datagram Protocol (UDP), Real-Time Transport Protocol (RTP), Transport Layer Security (TLS), Datagram TLS (DTLS), and others. In many works [16,17,18,19,20,21], bandwidth efficiency, reliability, energy consumption, and the security of these protocols have been studied. De Caro et al. [22] analyzed the MQTT and CoAP lightweight protocols designed for highly resource constrained environments. Qualitative and quantitative comparisons of these protocols have shown that CoAP, which is designed on top of UDP, is more efficient. Because video data streaming has the various requirements listed above, some packet loss is possible; therefore, video data streaming can be most effectively implemented using the UDP protocol at the transport layer.

Various schemes of the cryptographic source authentication are based on the hash-chain method. Yang et al. [23] proposed a source authentication scheme for the multicast based on a message recovery signature scheme. The proposed scheme ensures authentication, confidentiality, and integrity of source for the multicast, and provides the packet’s sequence number, which is important for streaming video. The scheme is tolerant to packet loss, however the amount of additional information for each transferred packet is large, approximately equal to the amount of useful information. Wang et al. [24] proposed a two-tier signature-hash (TTSH) stream authentication scheme to improve the video quality by reducing the authentication dependence overhead while protecting its integrity. The TTSH scheme achieves considerable gains in both authenticated video quality and energy efficiency. Perrig et al. [25] introduced a Timed Efficient Stream Loss-Tolerant Authentication (TESLA) broadcast authentication scheme. The TESLA scheme uses time and key series to authenticate the source of the data stream. However, the TESLA scheme is difficult to adapt for real-time streaming and does not provide non-repudiation of source. Each cryptography key should be used for a period; therefore, the system time of the sender and the receivers must be the same. Because the received packets can only be checked during the next period, there may be a delay of the data stream. We proposed in our previous work [26] the use of an energy efficient SSL protocol, which provides the most effective ratio between energy consumption and security of the data that is transmitted. Usman et al. [11] proposed a clustering-based technique for authenticating data streams. This energy-efficient data streaming approach authenticates the data streams and maintains the quality of transmitted data. The authentication scheme is two-step: (1) node authentication, and (2) secure transmission of the data stream through data authentication. Data packet authentication is based on crypto-hash tags, which connect each data packet with its former. Because it uses crypto-hash tags, the amount of transferred data is larger; consequently, there is an additional energy consumption cost of about 20%.

Wendzel et al. [27] reviewed the existing methods to create covert channels in various network and application protocols. Covert channels can be implemented in different layers of the TCP/IP protocol stack and can also be used for authentication of data sources and content [28,29,30]. To enhance network covert channels properties, protocol headers, so-called micro-protocols, are added to hidden payload in covert channels. Such protocol headers enable fundamental features such as reliability, dynamic routing, proxy capabilities, simultaneous connections, or session management for network covert channels—features which enrich communications to become more adaptive and stealthier. Wendzel et al. [31] provide the overview and categorization of micro-protocols. Xie et al. [29] proposed an identity authentication method based on the reverse usage of the network covert channel, where the packet intervals are exploited as the data carrier to transmit the identity tag. The authors implemented this method on the FTP platform. The data rate is considerably reduced in the covert timing channel. These experiments demonstrated that, depending on the channel noise, the data rate decreases from 28–47%. Islam et al. [30] proposed a technique for authenticating the geolocation of IoT devices using a covert channel. The proposed authentication technique is based on the Physical Unclonable Function (PUF) and ICMP covert channel.

Frączek et al. [32] introduced steganography methods, known as deep hiding techniques, that can be applied to any existing network steganography method to make it even more undetectable. Five different types of deep hiding techniques are discussed in the paper, including Steganogram Scattering, Steganogram Hopping, Carrier Modifications Camouflage, Inter-Protocol Steganography, and Multi-Level Steganography (MLS). All of these methods increase the undetectability properties of the existing methods by using mixes of different steganographic methods and/or carrying network protocols, scattering data between several sending hosts, and so forth. The concept of multilevel steganography is further discussed by Frączek et al. in their other work [33]. MLS uses two or more different steganographic methods simultaneously. The so called upper-level method is used as the carrier for the lower-level method. The lower-level steganography method is harder to detect even in cases where existence of the upper level method is discovered. The authors proposed various scenarios for the application of the MLS, in which the more secure lower-level method is used to transfer more sensitive data such as encryption keys, integrity, or authentication information for secret data carried by the upper-level method. The lower-level method could also be used to transfer parameter changes for the upper-level method to make it harder to detect (e.g., to change the upper-level method and cause Steganogram Hopping). Kesavan Gopal [34] proposed to embed a watermark—a unique device identifier or user-defined payload—at the beginning stage of digital video stream production. This method is independent of the protocol and semi-fragile in nature and is resistant to attacks with few packet losses in the network.

## 3. Lightweight Secure Streaming Protocol

We propose the use of a lightweight secure streaming protocol (LSSP) for the fog computing “Fog Node-End Device” layer. This protocol is intended for data streaming where a certain degree of packet loss is possible; for example, video streaming using resource-constrained devices and using bandwidth-constrained networks.

The main properties of the protocol are:

- Connectionless authenticated data streaming.
- Depending on the mode, the protocol can provide various security properties: authentication of source, authentication and integrity of data, confidentiality of data, and resilience to some data loss during transmission.
- Security properties of the data stream can be easily resumed after transmission interruptions without additional authentication steps from the sending or receiving parties.
- The protocol provides zero data overhead because all additional security information is embedded into the headers of modified UDP packets while the data fields of the UDP packets are left untouched.
- The protocol supports data stream broadcasting (multicasting).
- The protocol relies on simple security algorithms such as secure hash functions, HMACs and symmetric encryption, which could be easily implemented in fog end devices.

The protocol is based on the following techniques:

1. UDP transport with a modified packet headers [35] is used for the data stream transfer between the end device and fog node. Information embedded in the UDP header fields is used for data packet reordering and security checks.
2. The protocol provides continuous data source and data stream authentication [29] based on an end device authenticator [36], time stamps, secure hash functions, and hash based message authentication codes (HMACs).
3. Data confidentiality is provided using secure encryption algorithms that utilize secret keys and timestamping.
4. Redundant data, such as error correction codes [37,38,39] and checksums [40], could be used to provide additional resilience to data packet loss during transmission.

To achieve efficient and flexible secure communication in the proposed protocol, we define three security modes:

- Mode 1—data source authentication only.
- Mode 2—data source authentication and content integrity.
- Mode 3—data source authentication, content integrity, and confidentiality.

### 3.1. Modified UDP for Secure Video Streaming

The proposed lightweight secure streaming protocol (LSSP) uses modified UDP packets with authentication information embedded into UDP packet headers. The original structure of the normal UDP packet is preserved, but some fields are used differently. Authentication information—dynamic devices and video stream authenticators—are generated from the secure device identifier and time stamp using hash functions. Authentication information is then divided into several pieces and inserted into the UDP headers. Because the UDP doesn’t guarantee delivery, original ordering, or deduplication of packets, we inserted the numbers of the data stream segments and packets into the proposed protocol. Error correction codes may be used for restoring lost authentication information, however lost video stream packets aren’t restored.

Sender authentication and data integrity is achieved by adding a message authentication code digest into the transmitted data. The digest is calculated at the sending side using a secure device identifier (*sid*) and secure hash functions (*h*). Due to limitations of space available in the UDP header, video stream packets are grouped into data segments $s_{i}$. Each segment is assigned a sequence number i=0, 1, …. The length of all segments is n packets and depends only on the message authentication code algorithm and additional redundancy information (the error correction code) added to the data stream. All data packets $p_{i,j},\;j=0,\;1,\;\ldots ,\;n-1$ forming the same data segment $s_{i}$ are ordered and assigned the sequence number i. Segment number i and packet number j are sent with each data packet into the modified UDP header. The segment number and packet numbers are used at the receiving side to reorder packets when individual packets are received out of their original order.

**Figure 2:**
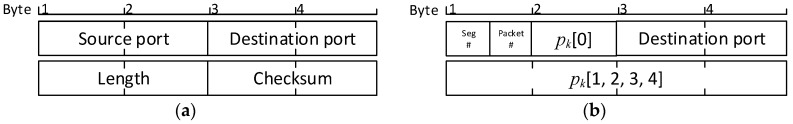
Comparison of a standard User Datagram Protocol (UDP) packet (**a**) and modified UDP packet (**b**) containing authentication data and segment and packet numbers.

The structure of the modified UDP packet is presented in Figure 2 and based on the concept of covert channel micro-protocols. Only the destination port field is left untouched from the original UDP packet header [35]. The second byte of the source port, packet length, and checksum fields is used to store five bytes of authentication data. The first byte of the source port is divided into two 4-bit nibbles. The first nibble is used to store segment number i, and the second nibble stores the sequential number of the packet in the current segment.

For example, if the HMAC-SHA1 algorithm is used for authentication, then the length of the corresponding data segment is n=4+1 data packets, as the length of the SHA1 based digest is only 160 bits, which fits into four packets’ headers with an additional packet used for checksum, using the XOR error correction code.

The selection of UDP header fields was based on the following assumptions:

The source port is not an important field as the sending device is identified by the destination port and authenticated using a different method;

The length of the UDP data is essentially a redundant field as the length of the data could be calculated from the IP header information;

The checksum field is not compulsory in the UDP header, moreover, data integrity is checked in data link layer. Additionally, data integrity could be checked in the LSSP protocol.

Free modification of some UDP header fields could lead to some issues in complex networks using routers, firewalls, etc., but the primary target of the LSSP protocol is communications between fog-nodes and end devices where only OSI Level 2 network infrastructure devices are used. Our observations show that modified UDP header fields do not cause any additional issues in the OS (Windows and Linux) network stack as long as low level network libraries are used (e.g., libpcap, winpcap [41], etc.)

### 3.2. The Generation of Secure Device Identifiers and Registration of End Devices

The first step of the protocol is registration of the new fog end device at the fog node and generation of the secure device identifier (sid), which is known only to the end device that streams data, and one (or more) of the fog nodes which receives data and checks its security properties. The secure device identifier is transferred to the fog nodes using a secure channel and is stored in the fog node. In order to register a new fog end device at the fog node, an initial secure channel must first be established. Since initial wireless interfaces are potentially insecure, an alternative secure communications channel is required. A direct wired connection between two components, e.g., using USB or ethernet, could provide sufficient protection. We propose using a wired connection for registration of the end device and wireless connection for further communications.

Authentication information (encryption keys in LSSP) is generated from the secure device identifier (sid). Therefore, this identifier must be unclonable, of good quality, generated truly randomly, contain sufficient entropy, be of sufficient length, and not be stored on the end device. For this purpose, physical unclonable functions (PUF) [39] are used, but a PUF is usually realized on special hardware. We have developed a secret encryption key generation algorithm by using the signature of the embedded system [36]. The proposed method effectively generates high-quality keys without any additional hardware and infrastructure cost, which is vital for devices with limited resources. We propose to use this algorithm for generating secure device identifiers.

Further, the algorithm for generating a secure device identifier (sid) by using the signature of the end devices is described in detail:

1. Create the set of signatures of the components of the end device $ES=\left\{es_{i}\right\},\;i=1,\;\ldots ,\;n$. The signature is created by applying the string concatenation of the Vendor ID ($cv_{i})$, Type ID ($ct_{i})$, Model ID ($cm_{i})$, and Serial Number ($csn_{i})$:
  (1) $$es_{i}=cv_{i}\|\;ct_{i}\|\;cm_{i}\|\;csn_{i}.$$
  In steps 2–6 a subset of the component signatures is created. These signatures will be used for computing of the end device signature.
2. Calculate the device’s embedded program header hash ph=h(p‖ psn).
3. Create the n×m matrix $MH=\left\{mh_{ij}\right\}$ from the bytes of the device’s embedded program header hash $mh_{ij}=eb\left(ph,\left(i-1\right)\times j+i\right)$, where *n* is the number of the end device’s signatures, and m=eb(ph,n) mod n.
4. Calculate the sum $s_{j}$ of the column elements in the matrix MH, $s_{j}=\sum_{i=1}^{n}mh_{ij},\;j=1,\;\ldots ,\;m$.
5. Create the index array of the component signatures $IND=\left\{ind_{j}\right\},\;\mathrm{where}\;ind_{j}=s_{j}\;mod\;n$ and delete repetitive indices, $ind_{j}\neq ind_{i},\;\forall \;i\in \left\{1\ldots j-1\right\}$ .
6. Create the subset of the component signatures $\tilde{ES}\subseteq ES,\;\tilde{es_{i}}=\;es_{j},\;\mathrm{where}\;j=ind_{k},\;\forall \;ind_{k}\in IND,\;k=1,\ldots ,\;m$, from which the end device signature will be created.
7. Create the signature of the end device $ss_{i}=sign\left(\tilde{ES}\right)$.
8. Generate the secret device identifier sid=fsid(ss, salt, iteration_count, key_length), where salt=eb(ph,n) mod n, $iteration\_count\;=count\left(\tilde{ES}\right)$.

### 3.3. LSSP Mode 1: Source Authentication

To provide data stream source authentication, all data packet headers carry a partial message authentication code digest and digest error correction code. Each packet’s UDP header includes a segment and packet number. The packet number is used to identify the correct order of the digest’s fragments, which are spread between different packets of the same segment. The sending party does not have to undertake any modifications or calculations on the transmitted data. The digest value does not depend on the data; data source authentication is provided by calculating:

1. $mac1_{i}=HMAC(sid,\;ts\;||\;i)$, where sid is the secure identifier of the source, ts is the current timestamp, and i is the number of the transmitted segment.
2. The digest is divided into fragments $p_{k}=submac\left(mac1_{i}\right),$ where $k=1\ldots m,\;m=lenght\left(mac1_{i}\right)/5$.
3. Calculating the $mac1_{i}$ error correction code: $ecc_{i}=fecc\left(p_{1}\ldots p_{k}\right)$, where fecc is the chosen error correcting function.
4. Inserting $p_{k}$ and $ecc_{i}$ into the UDP headers. Finally, all packets comprising the segment should be sent to the receiving party.

To authenticate the source of the data stream, the receiving party must collect all digest fragments $p_{k}$ of the same segment and combine them into $mac1_{i}$. If some packets were lost during transmission, lost fragments are restored using the error correction code. Additionally, the receiving party has to calculate its own version of the corresponding function to get $mac1_{r}$. If both values match, then the data source is authenticated.

### 3.4. LSSP Mode 2: Source and Content Authentication

To provide source and content authentication, the following procedure should be followed at the sending side:

1. For each new segment $s_{i}$ of the data packets, the new authentication key $k_{i}$ should be calculated using the following equation: $k_{i}=H\left(sid\;\left|\left|\;ts\;\right|\right|\;i\right)$, where sid is the secure identifier of the source, ts is the current timestamp, and i is the number of the transmitted segment.
2. All n data fragments comprising a full segment of packets should be collected and HMAC digest calculated on the data of all these packets using key $k_{i}$, $mac2=HMAC\left(k_{i},data\right)$, where data is the concatenation of the data of all packets comprising the current data segment.
3. The digest is divided into fragments $p_{k}=submac\left(mac1_{i}\right),$ where $k=1\ldots m,\;m=lenght\left(mac1_{i}\right)/5$.
4. Calculating the $mac1_{i}$. error correction code: $ecc_{i}=fecc\left(p_{1}\ldots p_{k}\right),$ where fecc is the chosen error correcting function.
5. Inserting $p_{k}$ and $ecc_{i}$ into the UDP headers. Finally, all packets comprising the segment should be sent to the receiving party.

The receiving party collects into memory all the packets comprising the whole segment of data and restores the sender’s digest value $mac2_{s}$ from the corresponding packets’ headers. If some fragments were lost, then missing fragments are restored using the error correction code. The receiver calculates the value of key $k_{i}$, and calculates its own version of the digest $mac2_{r}$ using the received data. If $mac2_{s}=mac2_{r}$ then all data packets comprising the whole data segment are unmodified, data integrity is intact, and the sender is authenticated.

### 3.5. LSSP Mode 3: Source Authentication, Content Authentication, and Confidentiality

To further enhance the security properties of the proposed protocol, it is possible to use symmetrical encryption to ensure confidentiality of the data. In this variation, source authentication and content integrity are ensured by using exactly the same procedure as in the Mode 2 modification of the algorithm. The only difference is that, after calculation of the digest, all data is encrypted using a symmetrical cipher (e.g., AES) in CBC mode.

At the sending party, each data packet is encrypted independently using a secret encryption key $ek_{i}$ and initialization vector $iv_{j}$, j=0, 1, …, n−1. The encryption key is the same for all packets of the i-th segment and is calculated using the following equation:

$ek_{i}=H\left(sid\;\left|\left|\;ts\;\right|\right|\;i\right)$, where sid is the secure identifier of the source, ts is the current timestamp, and H is the same secure hash function as used for the HMAC calculations. If the result is too long to be used as the key for the selected encryption algorithm, then it is truncated. On the other hand, a secure hash function should be chosen to provide a sufficient length of the hash result for use as an encryption key. For example, if AES256 is used for encryption, then at least SHA256 should be used for the HMAC calculations.

The initialization vector used for the CBC encryption mode is different for each data packet and calculated using the following equation:

(2) $$iv_{j}=H\left(sid\;\left|\left|\;i\;\right|\right|\;j\right).$$

This kind of security parameter derivation ensures that the receiving party can decrypt data even in cases where some packets of the data segment are missing, and reconstruction of the whole segment is impossible.

If additional resilience to data loss is required, protocol modification could be obtained by adding additional redundant data packets with error correction information. In this case, the error correction code should be calculated for the data packets.

## 4. Evaluation and Experiments

### 4.1. Qualitative Comparison

One of the advantages of the proposed protocol over DTLS is the simplicity of new device registration. If the DTLS protocol is used, then server and client authentication is performed during the handshake procedure using x.509 certificates. X.509 certificates should be generated, signed, and distributed to all devices in the network. This requirement imposes the need for proper management, storage, revocation, and so forth of all the issued certificates.

The LSSP protocol does not use any special stages to establish the new connection between server and client. In its simplest variation (Mode 1), no additional information is added to the data fields of the network packets, ensuring zero traffic overhead is used for the source authentication data. On the other hand, DTLS uses the handshake procedure which takes long time (especially in lossy network conditions) and requires a mutual exchange of messages in the correct order. If the handshake is frequent it can create a considerable amount of network traffic. Moreover, the data fields of each DTLS packet are carrying additional security and protocol information, which further increases the overall traffic overhead.

Resuming the LSSP protocol session after a long delay does not require any special steps. The data source is automatically authenticated after the first reception of the whole segment of data. DTLS requires a handshake procedure and renegotiation of the connection parameters after all long delays.

DTLS is a point to point protocol, which implies that each client has to make an individual connection to the receiving server. This eliminates DTLS from situations where multicast data translation is required. On the other hand, the proposed LSSP protocol could be successfully used in environments where several data-receiving servers are present. Moreover, the LSSP protocol does not require an explicit connection to the server—the transmitting device could sit on the network and translate data even if no servers are listening to it at any specific time.

The DTLS protocol does not provide any means to fight possible problems under non-ideal network conditions. Only the handshake stage of DTLS ensures correct data delivery if packet loss occurs in the lower levels of the network stack. The proposed LSSP protocol anticipates usage of ECC for authentication data and for the payload (arbitrary), thus providing higher resistance to data loss due to transmission errors in the underlying network stack.

On the negative side, when compared to the plain UDP or DTLS, the LSSP protocol requires more memory at the sending device, as the data of the whole segment must be collected and stored in memory before the calculations of the authentication data and ECC can begin. This memory overhead increases when message authentication codes with longer digests are used. For example, if HMAC-SHA1 is used, then memory requirements for data buffer increases five-fold compared to the plain UDP. LSSP protocol also introduces some additional latency to the network data stream, as it has to wait until the data comprising the whole segment is available. Latency increases if “slow” data stream is used. This issue could be mitigated by using smaller packets.

### 4.2. Performance Comparison

To evaluate the performance characteristics of the proposed method, a streaming client and receiving server were created. As the prototype for the fog “End Device”, the Raspberry Pi embedded computer (Model B, revision 2, BCM2835 CPU, 512 MB RAM) running “Raspbian GNU/Linux 9 (stretch)” was used. All performance measurements were performed at the sending device. The receiving party was a standard PC running Windows 10. The LSSP protocol was implemented in java using open source security libraries from Bouncy Castle [42]. To get low level access to UDP packet headers, the jnetpcap java library [43], providing an interface to the low level libpcap [41] and winpcap system libraries, was used. The java native implementation of the DTLS protocol provided by Bouncy Castle [44] was used in tests involving DTLS.

Three modifications of the LSSP protocol were implemented. Mode 1 implementation used five packet length segments and the HMAC-SHA1 authentication function for data source authentication. The headers of the first four packets of the segment carried a 160-bit digest value and the last packet carried a checksum of the first four parts of the digest. A simple XOR function was used to calculate the checksum value. Implementation of Mode 2 used the same message authentication function as Mode 1. The only difference was that HMAC-SHA1 was additionally calculated on the data fields in the first four packets of the five-packet segment. The last packet’s data field was filled with ECC values for the first four data packets. The XOR function was used to calculate both checksums. Finally, Mode 3 implementation additionally encrypted all data packets using a standard AES block cipher in CBC mode with a 128-bit key. In the following graphs these three modifications of the LSSP method are labeled as M1, M2, and M3.

To compare the performance characteristics of the proposed method we used a standard UDP protocol, which does not provide any security or source authentication, and two different variations of the DTLS protocol. The first variation of DTLS used the TLS_RSA_WITH_NULL_SHA ciphersuite, which does not provide any encryption, but authenticates the source and the data. As the security qualities of this variation are comparable to Mode 2 of the LSSP method, this DTLS variation is labeled as DTLS2 in the following charts. The second variation of DTLS used the TLS_RSA_WITH_AES_128_CBC_SHA ciphersuite and is labeled DTLS3 in following charts, because its security properties are comparable to Mode 3 of the LSSP method.

### 4.3. Experimental Results

**Figure 3:**
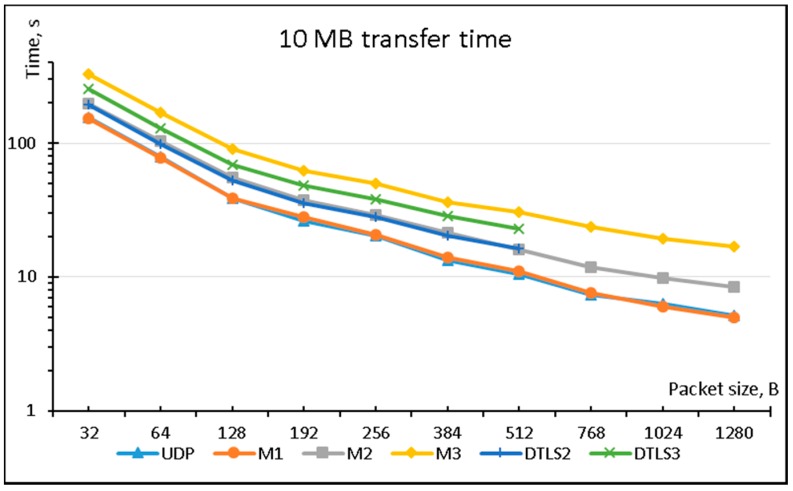
Comparison of time taken to transfer 10 MB of data using various protocols.

Figure 3 compares the performance of the six aforementioned protocols with respect to transmission time while transferring 10 MB of data using different packet lengths. The plain UDP is the best performer, but Mode 1 implementation of LSSP method is a very close competitor and additionally provides data source authentication. The difference in performance of plain UDP and M1 is caused by computational overhead introduced by additional calculations performed on the sending device. The total amount of plain data sent through the network interface is exactly the same in both cases, but time taken to send all the packets is slightly bigger in the case of M1. For example, it takes 10.5 s to send 10 MB in 512 B data packets using UDP and 11 s using M1.

Another interesting pair is M2 and DTLS2, which provide almost identical levels of performance and comparable properties of source and data authentication.

M3 is slower than DTLS3 because additional data packet containing ECC information for data is send in each data segment.

**Figure 4:**
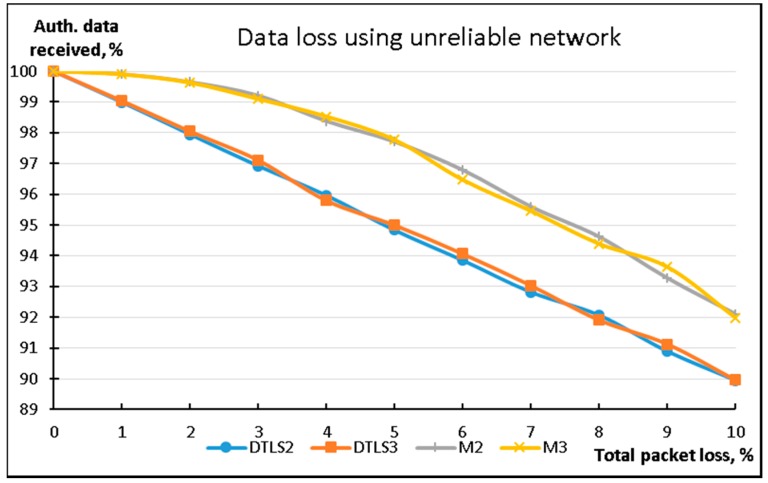
Comparison of data losses while using a non-ideal network infrastructure.

To evaluate the performance of the methods in non-ideal networks we used the NetEM (Network Emulation) tool, which allowed us to emulate various faulty conditions of network functionality. We used NetEM [45] on the data sending device (Raspberry Pi) to emulate random packet loss in the network infrastructure. On the receiving side all data packets were collected, and missing data packets were restored (if possible). The results of this experiment are presented in Figure 4.

During this experiment, only DTLS2, DTLS3, M2, and M3 were used. Ten MB of data was transferred from the streaming device using 256 B packets. Results show that both the M2 and M3 modifications of the LSSP method provide significantly less overall data loss in poor network conditions by using redundant data packets for missing data restoration at the receiving side.

**Figure 5:**
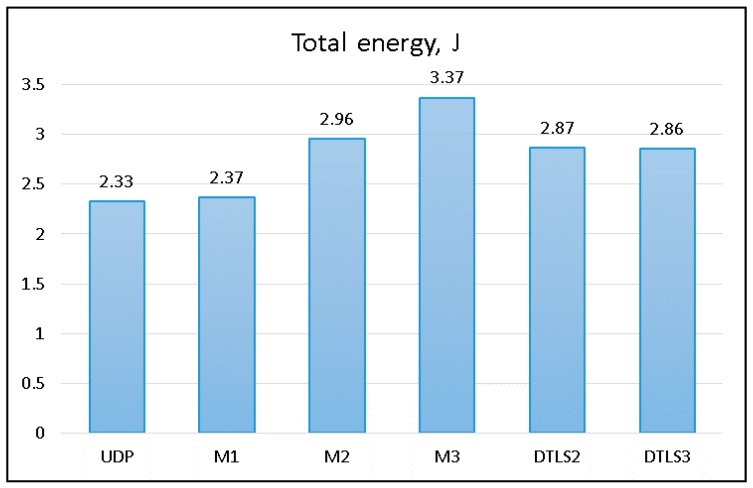
Energy consumption comparison.

To assess the energy efficiency of the proposed method, we measured total energy consumption of the streaming device (energy consumed by the USB WiFi adapter attached to the Raspberry Pi) while sending 10 MB of data using 256 B packets. The results are presented in Figure 5.

**Figure 6:**
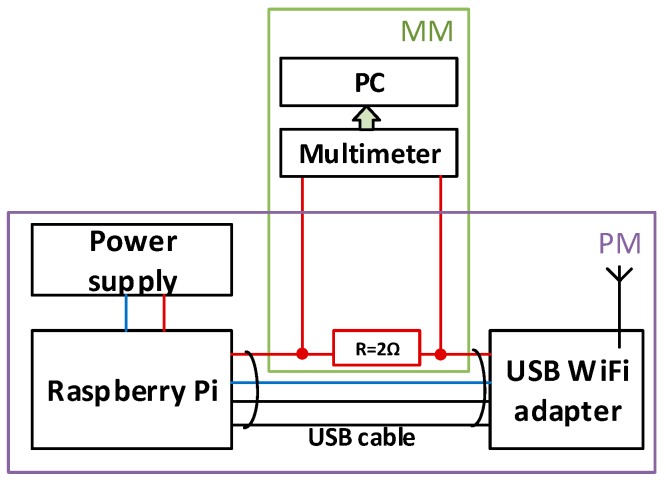
Energy consumption measuring. PS—power supply, PM—end device prototype module, MM—energy measuring module.

To measure the energy consumption in the WiFi adapter we used the arrangement presented in Figure 6.

Energy consumption was measured at the sending device (Digitus Wireless 150N USB adapter) using current shunt and bench multimeter. A personal computer was used to collect measured data and calculate total energy consumption during data transmission. Quiescent energy used to power the USB WiFi adapter was excluded from this evaluation.

DTLS and corresponding LSSP protocols were using the same security algorithms implemented using the same native Java cryptographic libraries. This ensures that power usage for encryption of the data is the same between different algorithms providing the same security level. The main objective of this experiment was to evaluate energy consumption differences while transmitting data.

**Figure 7:**
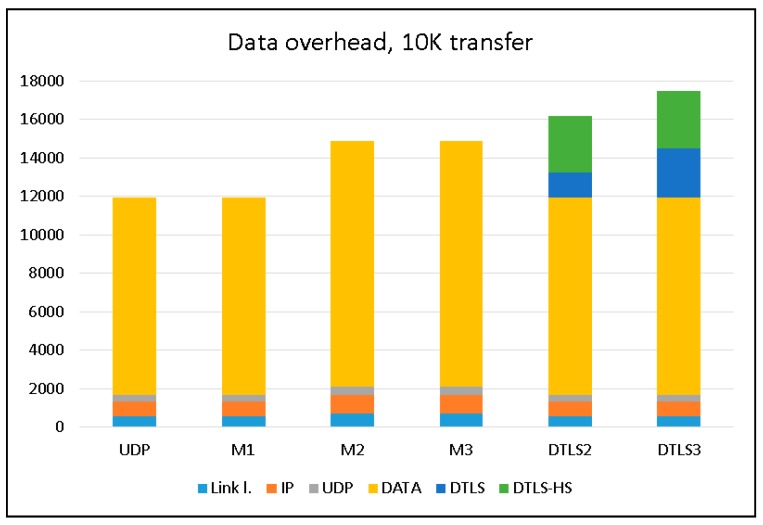
Data overhead comparison.

To evaluate the data overhead imposed by all protocols under evaluation, we transferred 10 K of data from the streaming device and used Wireshark to capture all packets at the receiving device, and then calculated the total sizes of the packets “on-wire”. The results are summarized in Figure 7. Only packets originating at the streaming device (client in the terms of DTLS) are considered in this chart. The handshake phase of the DTLS protocol imposes some traffic passed from the server to the client.

This chart clearly shows that the M1 modification of LSSP does not add any additional data compared to the most bandwidth effective plain UDP protocol. The M2 and M3 versions use an additional data packet to send ECC information for the data, so the total bandwidth used in these cases is exactly 5/4 of that used for UDP or M1. Both versions of DTLS use the handshake stage (labeled as DTLS-HS) and additional DTLS protocol related data in each data packet (labeled as DTLS).

## 5. Conclusions

In this paper, a new lightweight secure streaming protocol (LSSP) for the fog computing “Fog Node-End Device” layer was introduced. This protocol is intended to be used by resource-constrained devices for data streaming, where a certain degree of packet loss is possible (e.g., video streaming).

The proposed protocol uses a covert channel-inspired communication over UDP with data source authentication information embedded into the UDP packet headers. The three basic modes of the protocols we investigated provided data source authentication only, content and data source authentication, and content confidentiality with data source authentication. In non-ideal network conditions, when extensive data loss is probable, additional redundant data packets could be added to increase data transfer reliability. The protocol is suitable for use in situations where broadcasting and multicasting is required, does not require special stages for network session establishment between the client and server, and is resilient (to some extent) to data packet losses in the network infrastructure.

The experimental results show that the Mode 1 modification of the proposed protocol has almost the same performance, data overhead, and energy consumption characteristics as the plain UDP protocol, while at the same time providing data source authentication.

The Mode 2 and Mode 3 modifications, when used with additional redundant ECC packets, provide increased data transfer reliability compared to the DTLS protocol, with similar security properties. On the other hand, if the usage scenario requires frequent establishment of new connections, then the bandwidth overhead used in the DTLS protocol as well as the complex handshake procedure causes DTLS to be less bandwidth efficient, even in cases when additional ECC packets are used for the LSSP protocol.
